# The effects of etidronate on brain calcifications in Fahr’s disease or syndrome: rationale and design of the randomised, placebo-controlled, double-blind CALCIFADE trial

**DOI:** 10.1186/s13023-024-03039-7

**Published:** 2024-02-07

**Authors:** Birgitta MG Snijders, Gini Mathijssen, Mike JL Peters, Marielle H Emmelot-Vonk, Pim A de Jong, Susan Bakker, Heleen A Crommelin, Ynte M Ruigrok, Eva H Brilstra, Vera PM Schepers, Wilko Spiering, Evelien van Valen, Huiberdina L Koek

**Affiliations:** 1 Department of Geriatrics, University Medical Center Utrecht, Utrecht University, Utrecht, The Netherlands; 2 Department of Internal Medicine, University Medical Center Utrecht, Utrecht University, Utrecht, The Netherlands; 3 Department of Radiology, University Medical Center Utrecht, Utrecht University, Utrecht, The Netherlands; 4 Department of Physiotherapy, University Medical Center Utrecht, Utrecht University, Utrecht, The Netherlands; 5 Department of Clinical Pharmacy, University Medical Center Utrecht, Utrecht University, Utrecht, The Netherlands; 6 Department of Neurology, University Medical Center Utrecht, Utrecht University, Utrecht, The Netherlands; 7 Department of Genetics, University Medical Center Utrecht, Utrecht University, Utrecht, The Netherlands; 8 Department of Rehabilitation, Physical Therapy, Science & Sports, UMC Utrecht Brain Center, University Medical Center Utrecht, Utrecht University, Utrecht, The Netherlands

**Keywords:** Fahr’s disease, Fahr’s syndrome, Primary Familial Brain Calcification, Etidronate, Cognitive functioning, Brain calcifications, Randomised-controlled trial

## Abstract

**Background:**

Fahr’s disease and syndrome are rare disorders leading to calcification of the small arteries in the basal ganglia of the brain, resulting in a wide range of symptoms comprising cognitive decline, movement disorders and neuropsychiatric symptoms. No disease-modifying therapies are available. Studies have shown the potential of treatment of ectopic vascular calcifications with bisphosphonates. This paper describes the rationale and design of the CALCIFADE trial which evaluates the effects of etidronate in patients with Fahr’s disease or syndrome.

**Methods:**

The CALCIFADE trial is a randomised, placebo-controlled, double-blind trial which evaluates the effects of etidronate 20 mg/kg during 12 months follow-up in patients aged ≥ 18 years with Fahr’s disease or syndrome. Etidronate and placebo will be administered in capsules daily for two weeks on followed by ten weeks off. The study will be conducted at the outpatient clinic of the University Medical Center Utrecht, the Netherlands. The primary endpoint is the change in cognitive functioning after 12 months of treatment. Secondary endpoints are the change in mobility, neuropsychiatric symptoms, volume of brain calcifications, dependence in activities of daily living, and quality of life.

**Results:**

Patient recruitment started in April 2023. Results are expected in 2026 and will be disseminated through peer-reviewed journals as well as presentations at national and international conferences.

**Conclusions:**

Fahr’s disease and syndrome are slowly progressive disorders with a negative impact on a variety of health outcomes. Etidronate might be a new promising treatment for patients with Fahr’s disease or syndrome.

**Trial registration:**

ClinicalTrials.gov, NCT05662111. Registered 22 December 2022, https://clinicaltrials.gov/ct2/show/NCT01585402.

## Background

Fahr’s disease and syndrome are rare disorders leading to calcification of the small arteries in the basal ganglia of the brain, resulting in a wide range of symptoms comprising cognitive decline, movement disorders (ataxia, dystonia, Parkinsonism), neuropsychiatric symptoms (depression, anxiety, psychosis) and various other signs (migraine, speech disorders, pain, seizures) [1]. The age of onset varies, but generally is between 30 and 50 years with a gradual progression of symptoms [1]. Symptomatic patients have an increased risk for dependence in activities of daily living and impaired quality of life [2]. Fahr’s disease is also known as Primary Familial Brain Calcifications (PFBC). Thus far, pathogenic mutations in six genes have been identified causing PFBC: four dominantly-inherited genes (SLC20A2, XPR1, PDGFB and PDGFRB) and two recessively inherited genes (MYORG and JAM2) [3]. Also, a number of other causes of calcifications of the basal ganglia are known, including for example infectious, metabolic/endocrine, and toxic diseases [4]. When the calcifications are secondary to another cause, the term Fahr’s syndrome is used. Initially, Fahr’s disease was considered a rare disease with a prevalence of 4.5 per 10,000, however, new genetic and imaging studies suggest a prevalence of 2.1 to 6.6 per 1,000 [5, 6]. This implies underdiagnosis probably due to heterogeneity and even lack of symptoms and limited familiarity among patients and physicians.

Treatment of Fahr’s disease or syndrome focusses on symptom management, for example with antidepressants or physiotherapy. To date, no disease-modifying drugs are available. The precise pathophysiologic mechanism that results in the calcifications of small arteries remains to be established [7]. Several studies have shown the potential of treatment of vascular calcifications with bisphosphonates, for example using etidronate [8–13]. Etidronate is a first-generation bisphosphonate that can be used for the treatment of bone diseases associated with excessive bone resorption, like osteoporosis [14]. Bisphosphonates are known as structural analogues of inorganic pyrophosphate (PPi), which is known to be the strongest inhibitor of vascular calcifications [7]. First-generation bisphosphonates have a closer chemical structure to PPi in comparison to more modern bisphosphonates [14]. Therefore, researchers have taken a special interest in studying the effects of etidronate to treat pathological calcifications [8–15]. For example, two clinical trials performed in haemodialysis patients showed that etidronate suppressed or even reduced vascular calcification [11, 12]. A randomised, placebo-controlled trial in patients with Pseudoxanthoma Elasticum (PXE), a rare hereditary systemic calcification disorder, demonstrated that etidronate can effectively halt the calcification process [8, 9]. Furthermore, etidronate has been used for prenatal therapy in pregnant women in whom a diagnosis of Generalised Arterial Calcification of Infancy (GACI) was suspected, aiming to decrease perinatal mortality [10, 16]. 

Against this background bisphosphonate therapy might be a potential treatment for patients with Fahr’s disease or syndrome as well, with etidronate being most promising. The primary objective of this randomised, placebo-controlled, double-blind trial is to determine whether etidronate treatment during 12 months can halt or attenuate deterioration of cognitive functioning in patients with Fahr’s disease or syndrome. Secondary objectives are to determine the effects of etidronate on mobility, neuropsychiatric symptoms, volume of brain calcifications, dependence in activities of daily living, and quality of life. This paper describes the design and rationale of the CALCIFADE trial.

## Methods

### Study design

The CALCIFADE trial (an acronym for ‘treatment of CALCIfications in FAhr’s DiseasE') is a randomised, placebo-controlled, double-blind trial comparing the effects of etidronate versus placebo in patients with Fahr’s disease or syndrome. The study is conducted at the outpatient clinic of the University Medical Center Utrecht (UMCU), which is an academic hospital in The Netherlands. This is the center in which most Dutch patients with Fahr’s disease and syndrome are seen. Patient recruitment started in April 2023. This study has received funding from the Dutch Brain Foundation (in Dutch: Hersenstichting, grant number DR-2021-00387).

The study is registered at the Dutch Central Committee on Research Involving Human Subjects (CCMO) (registration number NL83131.041.22), ClinicalTrials.gov (registration number NCT05662111), and EudraCT-database (registration number 2022-003299-17).

### Participants

Patients will be recruited at the outpatient clinic of the UMCU and by recruitment through (social) media, for example using the UMCU website, the Dutch Fahr society for patients, newsletters, yearly patient days, and conferences. In total, 98 patients will be recruited (a sample size calculation is included in the ‘Statistical analyses’ section).

In order to be eligible to participate in this study, a subject must meet the following inclusion criteria: (1) age of 18 years or over, (2) clinical diagnosis of Fahr’s disease or syndrome. No international accepted diagnostic criteria for Fahr’s disease or syndrome exist yet. It is diagnosed mostly based on the clinical presentation. For the present study the following criteria are used: (a) presence of clinical symptoms consistent with a clinical diagnosis of Fahr’s disease or syndrome and (b) bilateral calcifications of the basal ganglia as seen on a CT-scan of the head, which cannot be (solely) due to the natural ageing process. Furthermore, the next criteria are supportive for the clinical diagnosis of Fahr’s disease, but not mandatory: (c) a positive family history for Fahr’s disease, and (d) the presence of a (likely) pathogenic variant in one of the PFBC-related genes.

Exclusion criteria are: (1) unable or unwilling to sign an informed consent, (2) severe renal impairment (estimated glomerular filtration rate (eGFR) of < 30 ml/min/1.73m2 calculated using CKD-EPI equation), (3) contraindication to receiving oral medication (for example severe dysphagia), (4) known abnormality of the oesophagus that would interfere with the passage of the drug (for example oesophageal strictures or achalasia), (5) known sensitivity to etidronate, (6) pregnancy, women with an active pregnancy wish < 1 year, or women who are breastfeeding at the time of inclusion, (7) inability to undergo a Dutch neuropsychological assessment (for example, non-fluent Dutch speakers or severe visual, hearing or motor impairment), (8) any other medical or social condition that puts the subject at risk of harm during the study or might adversely affect the interpretation of the study data, (9) use of bisphosphonates during the last 5 years, (10) hypocalcaemia (calcium < 2.20 mmol/L), and 11) 25-OH vitamin D deficiency < 35 nmol/L. After correction of hypocalcaemia or vitamin D deficiency, a participant is again suitable for participation.

Subjects can leave the study at any time for any reason if they wish to do so without any consequences. The investigator can decide to withdraw a subject from the study for urgent medical reasons. Individuals withdrawn from the study (for any reason) will be replaced.

### Intervention

Patients will be randomised to either daily, oral, etidronate 200 mg capsules 20 mg/kg for two weeks on followed by ten weeks off during 12 months (4 cycles in total), or an identical product without the active pharmacological substance (placebo) in the same cyclical regimen. The number of capsules will be rounded up to the nearest quantity that is divisible to 400 mg. We are expecting to switch to 400 mg capsules during the study period when they can be manufactured in order to further enhance patient medication adherence, as the number of capsules prescribed per day will be reduced. Patients will be instructed to take their study medication on an empty stomach in the morning with a full glass of water and sit upright for 30 min afterwards.

Etidronate is a known and safe drug, generally well tolerated and side effects are mild. The most reported side effects are transient musculoskeletal pain and gastro-intestinal side effects like diarrhoea, dyspepsia and oesophageal erosions. However, a systematic review revealed that there was no additional risk for gastro-intestinal problems associated with bisphosphonate use, if administered properly [17]. A rare side effect is osteonecrosis of the jaw. This side effect has been reported mostly in patients with cancer receiving chemotherapy and corticosteroids [18]. Theoretically, etidronate could have an effect on calcium homeostasis and bone turnover. Therefore, calcium, phosphate and vitamin D will be included as safety measures [19]. The relatively high dosage of etidronate used in this study protocol is equal to the dosage used in studies in patients with other rare hereditary calcification disorders [8, 9, 15]. 

Etidronate and the matching placebo will be produced by Pharmacy A15, the Netherlands. The products will be packaged and shipped to the clinical trial pharmacy of the UMCU. At every follow-up visit participants will be asked to take any redundant product and the empty packaging material, to monitor drug adherence.

Randomisation will be performed 1:1 using computer-generated block randomisation with variable block sizes by the Clinical Drug Research Unit of the UMCU. Both patients and researchers will be blinded for allocation arm during treatment. Deblinding of study medication of individual participants is only possible in the case of a medical emergency in which deblinding has consequences for the medical treatment of the participant.

### Outcome measures

The primary endpoint is the change in cognitive functioning in patients with Fahr’s disease or syndrome treated with etidronate compared to placebo between baseline and 12 months after baseline. Cognitive functioning is measured by means of a standardized neuropsychological test battery covering global cognitive functioning and three cognitive domains; (1) memory, (2) attention and speed of information processing, and (3) executive functioning.

Global cognitive functioning is assessed with the Dutch version of the Montreal Cognitive Assessment (MoCA) [20]. Memory with the Dutch Rivermead Behavioral Memory Test Stories immediate and delayed recall [21, 22], and with the Rey Complex Figure test immediate and delayed recall [23]. Attention and speed of information processing with the third edition of the Wechsler Adult Intelligence Scale (WAIS-III) Digit Span Forward [24], Trail Making Test A [25], and Stroop I and II [26]. Executive functioning with the WAIS-III Digit Span Backward, Trail Making Test B and Stroop III, semantic [27] and letter [28] fluency and social cognition with the FEEST [29]. A certified neuropsychologist will perform these tests.

An observational pilot study at our center (data not published yet) showed that patients with Fahr’s disease or syndrome (*n* = 26) often have deterioration of global cognitive functioning (mean MoCA 24.6 (out of 30); 32% of patients MoCA < 24). Cognitive domains that were most often impaired are memory (35% of patients), attention and speed of information processing (46% of patients), and executive functioning (42% of patients).

Secondary endpoints are the change in mobility, neuropsychiatric symptoms, volume of brain calcifications, dependence in activities of daily living, and quality of life in patients with Fahr’s disease or syndrome treated with etidronate compared to placebo between baseline and 12 months after baseline. Mobility symptoms are assessed with the condensed version of the Balance Evaluation Systems Test (Mini-BESTest) [30, 31], which is a composite test of gait and balance, by the research physician, and Unified Parkinson’s Disease Rating Scale (UPDRS) part III (motor examination) [32] by a physiotherapist. Neuropsychiatric symptoms are assessed with the Neuropsychiatric Inventory [33] by the research physician and with the Geriatric Depression Scale-15 [34, 35] by the neuropsychologist. Daily functioning is assessed with the Katz-15 scale [36] and quality of life with the 36-item Short Form Health Survey (SF-36) [37] by the research physician.

To determine the change in brain calcification volume, participants will be scanned on the IQon CT or CT7500 (Philips Healthcare) from skull base to vertex. Tube voltage will be 120 kVp and the tube current will range depending on patient size. Only non-contrast CT will be used to slice thickness < 1 mm. Images will be visually assessed in bone setting (centre: 300 Hounsfield Units– width: 1600 Hounsfield Units). Volume of the calcifications in the brain will be measured with standard software (Philips Intellispace Portal v11). Calcifications are defined as hyperdense lesions with a density above 130 Hounsfield Units. Care will be taken that all patients are scanned on the same scanner before and after treatment for all their investigations so that interscan differences can be avoided. In addition to volume measurements, a CT-based calcification score will be calculated as proposed by Nicolas et al. [38] Calcification is graded from 0 (no calcification) to 5 (serious and confluent) in specific locations of the brain; lenticular, caudate, thalamus nuclei, subcortical white matter, cortex, cerebellar hemispheres, vermis, midbrain, pons, and medulla. The total calcification score (ranging from 0 to 80) is obtained by adding all location-specific points. [38].

### Data collection

Table 1 shows an overview of data collection during the study period. Most data collected during the study period is part of standard of care at the outpatient clinic of the UMCU, as indicated in Table 1. At baseline, data will be collected on past medical history, family history, and medication use. A physical examination, laboratory tests, a CT-scan of the brain and tests to evaluate cognitive functioning, mobility, neuropsychiatric symptoms, dependence in activities of daily living, and quality of life will be performed at baseline and after 12 months of follow-up. If a patient drops out prematurely, an exit visit will be scheduled if possible. Additional safety blood analysis will be done at six months for the purpose of this study. Table 2 provides an overview of laboratory tests performed during the study period. Extensive infectious disease serology is performed at baseline to evaluate the presence of any other causes of brain calcifications (Fahr’s syndrome) and is part of standard of care. If infectious disease serology has already been performed in a patient in the last recent years, the research physician will decide whether these tests must be repeated at baseline. At three and six months, patients will be scheduled for telephone consultations for evaluation of treatment compliance and side effects. Researchers will be blinded during data collection.

**Table 1:** Overview of data collection during study period

	Screening		Treatment period						
	Visit 1	Visit 2	Cycle 1^α^	TC 1	Cycle 2^α^	TC 2	Cycle 3^α^	Cycle 4^α^	Completion visit
Weeks of study drug exposure	N/A	N/A	Week 0–12	Week 12	Week 13–24	Week 24	Week 25–36	Week 37–48	Week 49
Informed consent	X								
Medical history	X*								
Medication review	X*								
Physical examination	X*								X*
Laboratory measurements	X*	(X)^β^				X			X*
CT-scan brain	X*								X*
Neuropsychiatric assessment	X*								X*
Cognitive assessment	X*								X*
Mobility assessment	X*								X*
ADL assessment	X*								X*
Quality of Life assessment	X*								X*
Randomisation		X							
Administer study drug			X		X		X	X	
Adverse event monitoring	X	X	X	X	X	X	X	X	X

Abbreviations: TC = telephone consultation, ADL = activities of daily living

^α^ Each cyclus starts with etidronate 20 mg/kg for two weeks on and ten weeks off or placebo for two weeks on and ten weeks off

^β^ After correction of a hypocalcaemia or vitamin D deficiency, a participant might be eligible for inclusion again

* Activities marked with an asterisk are considered part of standard of care

**Table 2:** Overview of laboratory measurements during study period

	Screening		Treatment period						
	Visit 1	Visit 2	Cycle 1^α^	TC 1	Cycle 2^α^	TC 2	Cycle 3^α^	Cycle 4^α^	Completion visit
Weeks of study drug exposure	N/A	N/A	Week 0–12	Week 12	Week 12–24	Week 24	Week 25–36	Week 37–48	Week 49
Calcium	X*	(X)^β^				X			X*
Magnesium	X*					X			X*
Phosphate	X*					X			X*
Aluminum	X*								X*
Creatinine (and eGFR)	X*					X			X*
ASAT	X*					X			X*
ALAT	X*					X			X*
Albumin	X*	(X)^β^				X			X*
CRP	X*								X*
Complete blood count	X*								X*
ESR	X*								X*
Ferritin	X*								X*
PTH	X*								X*
25-OH-vitamin D	X*	(X)^β^				X			X*
Copper	X*								
Zinc	X*								
Tuberculosis (Quantiferon)	X*								
Brucella antibodies	X*								
HIV 1/2 antibodies p24 antigen	X*								
Rubella IgG & IgM	X*								
CMV QDNA	X*								
Human herpesvirus type 6 QDNA	X*								
Human herpesvirus type 8 DNA	X*								

Abbreviations: TC = telephone consultation, eGFR = estimated glomerular filtration rate, ASAT = aspartate transaminase, ALAT = alanine transaminase, CRP = C-reactive protein, ESR = erythrocyte sedimentation rate, PTH = parathyroid hormone, HIV = human immunodeficiency virus, Ig = immunoglobulin, CMV = cytomegalovirus, (Q)DNA = (quantitative)deoxyribonucleic acid

^α^ Each cycle starts with etidronate 20 mg/kg for two weeks on and ten weeks off or placebo for two weeks on and ten weeks off

^β^ After correction of a hypocalcaemia or vitamin D deficiency, a participant might be eligible for inclusion again

* Activities marked with an asterisk are considered part of standard of care

Castor will be used as electronic data capture system. Digital files will be stored in a secured Research Folder Structure of the UMCU. Data monitoring will be performed by an independent external party (Julius Clinical). Comprehensive data management and monitoring plans were drafted and are available from the corresponding author upon request.

### Statistical analyses

Researchers will be blinded during data analysis. Descriptive data will be presented as categorical (n, %), normally distributed continuous (mean ± standard deviation), or non-normally distributed continuous variables (median, interquartile range), as appropriate. A relatively small trial as the CALCIFADE trial is subject to a greater chance of imbalance in baseline characteristics than larger trials. Currently, covariate adjustment is a standard statistical approach in randomised-controlled trials [39]. Therefore, adjustment for baseline covariates will be performed in the primary/main analysis of the primary endpoint, as appropriate. Both the unadjusted and adjusted effects will be analysed and presented.

For the primary endpoint, composite Z-scores will be calculated for (1) global cognitive functioning, (2) memory, (3) attention and speed of information processing, and (4) executive functioning using normative data form the Advanced Diagnostics Infrastructure [40]. The differences in change in cognitive functioning between baseline and 12 months will be compared between the intervention and placebo groups and will be tested using multivariate (linear regression) analyses using as covariates baseline composite Z-scores and limited other relevant prognostic factors (as will be finalized in the statistical analysis plan before database lock). The mean treatment effect (difference between treatment and placebo) with its 95% confidence intervals will be reported. Etidronate therapy is considered effective if the mean treatment effect is significant (*p* < 0.05) and clinically relevant. A clinically relevant treatment effect is defined as a difference of ≥ 0.3 points in the mean change from baseline for the composite Z-scores for global cognitive functioning and/or one or more cognitive domains between the treatment and placebo group. As there are no published data on the natural course of neuropsychological functioning of patients with Fahr’s disease, the 0.3 points difference of the composites was based on populations with similar neuropsychological profiles as we suspect in Fahr’s disease: Parkinson’s disease and Chronic Solvent induced Encephalopathy, and using a comparable neuropsychological assessment as this study [41–43]. 

For the secondary endpoints, the same strategy will be used as for the primary endpoint. Missing data will be handled by multiple imputation, where appropriate. A subgroup analysis will be performed in patients with confirmed genetic mutations directly affecting the (inorganic) phosphate (Pi) homeostasis (SLC20A2, XPR1 and PDGFRB). Studies in animals have shown that the Pi/PPi ratio is a key factor in the development of mineralisation [44–46]. SLC20A2, XPR1 and PDGRB play an important role in phosphate transport and a mutation in one of these genes results in accumulation of Pi [7]. Elevated Pi levels are associated with reduced rates of PPi accumulation, which is known as a key inhibitor of vascular calcification [45]. As etidronate is a molecular homologue of PPi, we hypothesise patients with a mutation in one of these genes may potentially benefit more from etidronate treatment [7]. 

An interim analysis of safety measures will be performed by the Data Safety Monitoring Board of the UMCU. This interim analysis will be performed on side effects, laboratory values and CT-scans of the brain when 25%, 50%, 75%, and 100% of included patients has reached a follow-up of 6 or 12 months. If anything occurs, on the basis of which it appears that the disadvantages of participation may be significantly greater than was foreseen in the research proposal, the study will be suspended pending further review by the Medical Ethical Review Committee, except insofar as suspension would jeopardise the subjects’ health.

#### Sample size calculation

In total, a sample size of 88 patients (44 treatment and 44 placebo) is needed to detect a clinically relevant difference of 0.3 points in the mean change from baseline for the composite neuropsychological test score between the treatment and placebo group, assuming a standard deviation of 0.5, with a power of 80% and a two-sided alpha of 0.05. With an expected dropout rate of 10% during the course of the trial, inclusion of 98 patients is required.

All statistical analyses will be performed using IBM SPSS Statistics, version 27.0.

### Ethical considerations

The study will be conducted according to the principles of the Declaration of Helsinki (Fortaleza, Brazil, October 2013) and in accordance with the Dutch Medical Research Involving Human Subjects Act (WMO) and the requirements of Good Clinical Practice (GCP). This study has received ethical approval from the Dutch Medical Ethical Review Committee NedMec (registration number 22-1005/Gm-G) and CCMO. Written informed consent will be obtained from all participants by the research physician.

## Results

Recruitment of participants started in April 2023. Results are expected in 2026 and will be disseminated through peer-reviewed journals as well as presentations at national and international conferences.

## Discussion

Fahr’s disease and syndrome are slowly progressive disorders with a negative impact on a variety of health outcomes. Currently, no disease-modifying therapies are available.

Only case studies of modest quality have been published previously regarding bisphosphonate use in patients with brain calcifications. In 1998, a case report was published reporting the use of etidronate in a 59 year old man with PFBC, which improved speech and gait, but did not affect his spasticity, dystonia, ataxia, or amount of cerebral calcification quantified by CT-scan [47]. In 2006, another study was published regarding etidronate use in two patients with brain calcifications: (1) an 8 year old boy who received chemotherapy and a bone marrow transplantation for acute myelogenous leukaemia and had progressive cerebral calcifications, who developed symptoms of headaches and seizures, and (2) a 45 year old woman with a calcified cavernous haemangioma who had symptoms of multiple seizure types. Both patients showed improvement of symptoms when treated for 6 months, but no change in cerebral calcifications was observed after 12 months [48]. The most recent study (published in 2016) described a case series of 7 patients with primary brain calcifications treated with alendronate. In some cases symptoms improved, especially among younger patients. However, there were no changes in cerebral calcifications [49]. 

The positive effect of bisphosphonate therapy in other hereditary arterial calcification syndromes, including PXE and GACI, combined with the few case reports of etidronate or alendronate use in patients with brain calcifications, suggests that etidronate might be a new promising treatment for patients with Fahr’s disease or syndrome.

## Conclusion

The CALCIFADE trial is a randomised, placebo-controlled, double-blind trial which will evaluate the effect of etidronate on cognitive functioning, mobility, neuropsychiatric symptoms, volume of brain calcifications, dependence in activities of daily living, and quality of life in patients with Fahr’s disease or syndrome during 12 months follow-up. To our knowledge, the CALCIFADE trial will be the first trial evaluating a potential therapy in patients with Fahr’s disease or syndrome.

## Data Availability

The dataset generated during the current study will not be made publicly available due to the fact that Fahr’s disease or syndrome are rare conditions, which makes it possible to trace back data to an individual person and therefore compromises individual privacy. The dataset is available from the corresponding author on reasonable request.

## Abbreviations

ACDC: Arterial Calcification due to CD73 deficiency
CCMO: Central Committee on Research Involving Human Subjects (in Dutch: Centrale Commissie Mensgebonden Onderzoek)
GACI: Generalised Arterial Calcification of Infancy
GCP: Good Clinical Practice
Mini-BESTest: Balance Evaluation Systems Test, condensed version
MoCA: Montreal Cognitive Assessment
PFBC: Primary Familial Brain Calcifications
Pi: Inorganic phosphate
PPi: Inorganic pyrophosphate
PXE: Pseudoxanthoma Elasticum
SF-36: 36-item Short Form Health Survey
UPDRS: Unified Parkinson’s Disease Rating Scale
UMCU: University Medical Center Utrecht
WAIS-III: Wechsler Adult Intelligence Scale, third edition
WMO: Medical Research Involving Human Subjects Act (in Dutch: Wet medisch-wetenschappelijk onderzoek met mensen)

## References

[1] Nicolas G, Charbonnier C, de Lemos RR, Richard AC, Guillin O, Wallon D (2015). Brain calcification process and phenotypes according to age and sex: lessons from SLC20A2, PDGFB, and PDGFRB mutation carriers. Am J Med Genet B Neuropsychiatr Genet.

[2] Donzuso G, Mostile G, Nicoletti A, Zappia M (2019). Basal ganglia calcifications (Fahr’s syndrome): related conditions and clinical features. Neurol Sci.

[3] Schottlaender LV, Abeti R, Jaunmuktane Z, Macmillan C, Chelban V, O’Callaghan B (2020). Bi-allelic JAM2 variants lead to early-onset recessive primary familial brain calcification. Am J Hum Genet.

[4] Saade C, Najem E, Asmar K, Salman R, El Achkar B, Naffaa L (2019). Intracranial calcifications on CT: an updated review. J Radiol Case Rep.

[5] Nicolas G, Charbonnier C, Campion D, Veltman JA (2018). Estimation of minimal disease prevalence from population genomic data: application to primary familial brain calcification. Am J Med Genet B Neuropsychiatr Genet.

[6] Chen S, Cen Z, Fu F, Chen Y, Chen X, Yang D (2019). Underestimated disease prevalence and severe phenotypes in patients with biallelic variants: a cohort study of primary familial brain calcification from China. Parkinsonism Relat Disord.

[7] Peters MEM, de Brouwer EJM, Bartstra JW, Mali W, Koek HL, Rozemuller AJM (2020). Mechanisms of calcification in Fahr disease and exposure of potential therapeutic targets. Neurol Clin Pract.

[8] Kranenburg G, de Jong PA, Bartstra JW, Lagerweij SJ, Lam MG, Ossewaarde-van Norel J (2018). Etidronate for Prevention of ectopic mineralization in patients with Pseudoxanthoma Elasticum. J Am Coll Cardiol.

[9] Bartstra JW, de Jong PA, Kranenburg G, Wolterink JM, Isgum I, Wijsman A (2020). Etidronate halts systemic arterial calcification in pseudoxanthoma elasticum. Atherosclerosis.

[10] Uitto J, Li Q, van de Wetering K, Varadi A, Terry SF (2017). Insights into pathomechanisms and Treatment Development in Heritable Ectopic Mineralization disorders: Summary of the PXE International Biennial Research Symposium-2016. J Invest Dermatol.

[11] Nitta K, Akiba T, Suzuki K, Uchida K, Watanabe R, Majima K (2004). Effects of cyclic intermittent etidronate therapy on coronary artery calcification in patients receiving long-term hemodialysis. Am J Kidney Dis.

[12] Ariyoshi T, Eishi K, Sakamoto I, Matsukuma S, Odate T (2006). Effect of etidronic acid on arterial calcification in dialysis patients. Clin Drug Investig.

[13] Koshiyama H, Nakamura Y, Tanaka S, Minamikawa J (2000). Decrease in carotid intima-media thickness after 1-year therapy with etidronate for osteopenia associated with type 2 diabetes. J Clin Endocrinol Metab.

[14] Drake MT, Clarke BL, Khosla S (2008). Bisphosphonates: mechanism of action and role in clinical practice. Mayo Clin Proc.

[15] Akhtar Ali S, Ng C, Votava-Smith JK, Randolph LM, Pitukcheewanont P (2018). Bisphosphonate therapy in an infant with generalized arterial calcification with an ABCC6 mutation. Osteoporos Int.

[16] Rutsch F, Boyer P, Nitschke Y, Ruf N, Lorenz-Depierieux B, Wittkampf T (2008). Hypophosphatemia, hyperphosphaturia, and bisphosphonate treatment are associated with survival beyond infancy in generalized arterial calcification of infancy. Circ Cardiovasc Genet.

[17] Cryer B, Bauer DC. Oral bisphosphonates and upper gastrointestinal tract problems: what is the evidence? Mayo Clin Proc. 2002;77(10):1031-43. 10.4065/77.10.1031 12374247

[18] Faiman B, Pillai AL, Benghiac AG (2013). Bisphosphonate-related osteonecrosis of the jaw: historical, ethical, and legal issues associated with prescribing. J Adv Pract Oncol.

[19] Watts NB, Chesnut CH 3rd, Genant HK, Harris ST, Jackson RD, Licata AA, et al. History of etidronate. Bone. 2020;134:115222. 10.1016/j.bone.2020.115222 31911206

[20] Nasreddine ZS, Phillips NA, Bedirian V, Charbonneau S, Whitehead V, Collin I (2005). The Montreal Cognitive Assessment, MoCA: a brief screening tool for mild cognitive impairment. J Am Geriatr Soc.

[21] Wilson B, Cockburn J, Baddeley A (1985). The rivearmead behavioural memory test.

[22] Van Balen HGG, Groot Zwaaftink AJM (1993). Rivearmead behavioural memory test. Nederlandse bewerking.

[23] Meyers JE, Meyers KR (1995). Rey Complex figure test and recognition trial. Professional manual.

[24] Wechsler D. Wechsler Adult Intelligence Scale. 3rd ed. San Antonio,TX Psychological Corporation; 1997.

[25] Reitan RM, Wolfson D (1985). The Halstead-Reitan Neuropsychological Test Battery.

[26] Hammes JGW (1971). De Stroop Kleur-Woord Test. Handleiding.

[27] Luteijn F, Barelds DPF (2004). GIT-2/ Groninger Intelligentie Test.

[28] Schmand B, Groenink SC, van den Dungen M (2008). [Letter fluency: psychometric properties and Dutch normative data]. Tijdschr Gerontol Geriatr.

[29] Young A, Perrett D, Calder A, Sprengelmeyer R, Ekman P (2002). Facial expressions of emotion—stimuli and tests (FEEST).

[30] Franchignoni F, Horak F, Godi M, Nardone A, Giordano A (2010). Using psychometric techniques to improve the balance evaluation systems Test: the mini-BESTest. J Rehabil Med.

[31] Keus SHJ, Munneke M, Graziano M (2016). De KNGF-richtlijn Parkinson: Nederlandse Uitgave Van European Physiotherapy Guideline for Parkinson’s Disease uit 2014.

[32] van Wegen EEH (2005). Klinimetrie Bij De Ziekte Van Parkinson: een praktische handleiding.

[33] De Jonghe JFMKM, Kalisvaart CJ (2003). Tijdschr Gerontol Geriatr.

[34] Yesavage JA, Brink TL, Rose TL, Lum O, Huang V, Adey M (1982). Development and validation of a geriatric depression screening scale: a preliminary report. J Psychiatr Res.

[35] Brink T, Yesavage JA, Lum O, Heersema PH, Adey M, Rose TL (1982). Screening tests for geriatric depression. Clin Gerontologist: J Aging Mental Health.

[36] Laan W, Zuithoff NP, Drubbel I, Bleijenberg N, Numans ME, de Wit NJ (2014). Validity and reliability of the Katz-15 scale to measure unfavorable health outcomes in community-dwelling older people. J Nutr Health Aging.

[37] Aaronson NK, Muller M, Cohen PD, Essink-Bot ML, Fekkes M, Sanderman R (1998). Translation, validation, and norming of the Dutch language version of the SF-36 Health Survey in community and chronic disease populations. J Clin Epidemiol.

[38] Nicolas G, Pottier C, Charbonnier C, Guyant-Marechal L, Le Ber I, Pariente J (2013). Phenotypic spectrum of probable and genetically-confirmed idiopathic basal ganglia calcification. Brain.

[39] Thompson DD, Lingsma HF, Whiteley WN, Murray GD, Steyerberg EW (2015). Covariate adjustment had similar benefits in small and large randomized controlled trials. J Clin Epidemiol.

[40] de Vent NR, van Agelink JA, Schmand BA, Murre JM, Consortium A, Huizenga HM (2016). Advanced Neuropsychological Diagnostics Infrastructure (ANDI): a normative database created from Control Datasets. Front Psychol.

[41] van Valen E, Wekking E, van Hout M, van der Laan G, Hageman G, van Dijk F (2018). Chronic solvent-induced encephalopathy: course and prognostic factors of neuropsychological functioning. Int Arch Occup Environ Health.

[42] Muslimovic D, Post B, Speelman JD, De Haan RJ, Schmand B (2009). Cognitive decline in Parkinson’s disease: a prospective longitudinal study. J Int Neuropsychol Soc.

[43] Broeders M, Velseboer DC, de Bie R, Speelman JD, Muslimovic D, Post B (2013). Cognitive change in newly-diagnosed patients with Parkinson’s disease: a 5-year follow-up study. J Int Neuropsychol Soc.

[44] Zhou X, Cui Y, Zhou X, Han J (2012). Phosphate/pyrophosphate and MV-related proteins in mineralisation: discoveries from mouse models. Int J Biol Sci.

[45] Prosdocimo DA, Wyler SC, Romani AM, O’Neill WC, Dubyak GR (2010). Regulation of vascular smooth muscle cell calcification by extracellular pyrophosphate homeostasis: synergistic modulation by cyclic AMP and hyperphosphatemia. Am J Physiol Cell Physiol.

[46] Orriss IR, Arnett TR, Russell RG (2016). Pyrophosphate: a key inhibitor of mineralisation. Curr Opin Pharmacol.

[47] Loeb JA (1998). Functional improvement in a patient with cerebral calcinosis using a bisphosphonate. Mov Disord.

[48] Loeb JA, Sohrab SA, Huq M, Fuerst DR (2006). Brain calcifications induce neurological dysfunction that can be reversed by a bone drug. J Neurol Sci.

[49] Oliveira JR, Oliveira MF (2016). Primary brain calcification in patients undergoing treatment with the biphosphanate alendronate. Sci Rep.

